# De novo triplication of 11q12.3 in a patient with developmental delay and distinctive facial features

**DOI:** 10.1186/1755-8166-6-15

**Published:** 2013-04-03

**Authors:** Toshiyuki Yamamoto, Mari Matsuo, Shino Shimada, Noriko Sangu, Keiko Shimojima, Seijiro Aso, Kayoko Saito

**Affiliations:** 1 Tokyo Women’s Medical University Institute for Integrated Medical Sciences, 8-1 Kawada-cho, Shinjuku-ward, Tokyo, 162-8666, Japan; 2 Institute of Medical Genetics, Tokyo Women’s Medical University, Tokyo, Japan; 3 Department of Pediatrics, Tokyo Women’s Medical University, Tokyo, Japan; 4 Department of Oral and Maxillofacial Surgery, School of Medicine, Tokyo Women’s Medical University, Tokyo, Japan; 5 Department of Pediatrics, Japanese Red Cross Medical Center, Tokyo, Japan

**Keywords:** Triplication, *STX5*, *CHRM1*, 11q12.3, Developmental delay

## Abstract

**Background:**

Triplication is a rare chromosomal anomaly. We identified a de novo triplication of 11q12.3 in a patient with developmental delay, distinctive facial features, and others. In the present study, we discuss the mechanism of triplications that are not embedded within duplications and potential genes which may contribute to the phenotype.

**Results:**

The identified triplication of 11q12.3 was 557 kb long and not embedded within the duplicated regions. The aberrant region was overlapped with the segment reported to be duplicated in 2 other patients. The common phenotypic features in the present patient and the previously reported patient were brain developmental delay, finger abnormalities (including arachnodactuly, camptodactyly, brachydactyly, clinodactyly, and broad thumbs), and preauricular pits.

**Conclusions:**

Triplications that are not embedded within duplicated regions are rare and sometimes observed as the consequence of non-allelic homologous recombination. The de novo triplication identified in the present study is novel and not embedded within the duplicated region. In the 11q12.3 region, many copy number variations were observed in the database. This may be the trigger of this rare triplication. Because the shortest region of overlap contained 2 candidate genes, *STX5* and *CHRM1*, which show some relevance to neuronal functions, we believe that the genomic copy number gains of these genes may be responsible for the neurological features seen in these patients.

## Background

**Figure 1:**
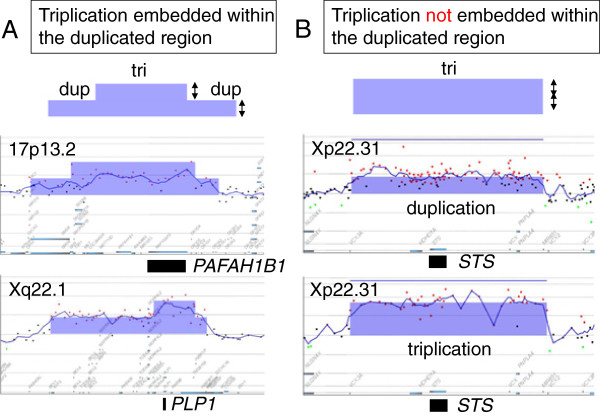
**Explanation of the 2 triplication patterns.** Each triplication pattern shown by Gene View of Agilent Genomic Workbench (Agilent Technologies) is schematically represented. The blue rectangles indicate chromosomal aberrations. The X- and Y- axes indicate the genomic location and a log_2_ ratio of the intensity, respectively. The log_2_ ratio of the triplicated region is 2-fold higher than that of the duplicated region. (A) The triplication embedded within the duplication region shows duplicated regions (dup) in both ends of the triplication (tri). The 2 examples at 17p13.2 and Xq22.1 include *PAFAH1B1* and *PLP1*, respectively. (B) The triplication not embedded within the duplicated region does not show any duplicated regions on either end of the triplication (tri) Two examples of duplication and triplication at Xp22.31 that include *STS* are shown. Both aberrations show the same range of the aberration. There are no duplicated regions around the triplicated segment. The black rectangles indicate the locations of the genes.

Triplication is a rare chromosomal aberration. Many chromosomal triplications that are visible by conventional karyotyping have been reported. Such chromosomal rearrangements are considered to be caused by abnormal meiotic chromosomal recombinations [1,2]. The introduction of chromosomal microarray testing allowed the identification of invisible small triplications. Although we have performed chromosomal microarray testing of >1,000 samples in our institution, only 3 invisible triplications have been identified to date (Figure 1). Thus, the frequency of such triplications is <0.3% in patients with developmental delay and/or congenital abnormalities.

When we analyzed genomic copy numbers in cases of triplication, the patterns of the results could be classified into 2 types according to genomic structures: (1) embedded within the duplicated segments and (2) not embedded within the duplicated segments (Figure 1). Two of the 3 triplications in our laboratory were embedded within the duplicated regions. A triplication identified at 17p13.3, including the platelet-activating factor acetylhydrolase 1b regulatory subunit 1 gene (*PAFAH1B1*) (a gene responsible for lissencephaly) was among them [3] (Figure 1). The triplicated segment was embedded within the duplicated regions, and fiber-fluorescence in situ hybridization (FISH) analysis confirmed that the triplicated segments were in a tandem orientation. A similar triplication of this region was also reported by Bi et al.; however, the nucleotide sequence of the breakpoints and/or inserted orientation was not analyzed [4]. Although breakpoint junctions have been analyzed in many cases with chromosomal rearrangements [5], it is challenging to map and sequence them in cases of copy number gains due to the existence of extra copies of the fragments, particularly in the case of autosomal chromosomes [6].

Another triplication embedded within the duplicated segments was identified in a patient with Pelizaeus-Merzbacher disease [7] (Figure 1), whose triplication, including the proteolipid protein 1 gene (*PLP1*), was caused by the mechanism of duplication-inverted triplication-duplication (DUP-TRP/INV-DUP). This mechanism was first reported by Carvalho et al. as the common mechanism in patients with recurrent triplication around the methyl CpG binding protein 2 gene (*MECP2*) located on Xq28 [8].

The third triplication identified in the Xp22.31 region was not embedded within the duplicated segments, and the aberration region was same as the duplication that is well-known as a popular copy number variation (CNV) (Figure 1). This triplication, including the steroid sylfatase gene (*STS*), was previously reported as not being embedded within the duplicated segments because the breakpoints were common among aberrations identified in individuals [6]. This finding indicated that non-allelic homologous recombination (NAHR) is mediated by low copy repeats (LCRs) surrounding the triplicated structures in this region, the orientation of the inserted triplicated segments remain unknown in those cases.

As mentioned above, a triplication embedded within a duplication is common in cases of random occurrences; however, triplications that are not embedded within duplicated regions may be caused by surrounding LCRs as a consequence of NAHR. In this study, we identified a de novo triplication that was not embedded within a duplication, in a patient with developmental delay and distinctive features. Herein, we discuss the genotype-phenotype correlation as well as the mechanism of chromosomal triplication.

### Case report

A 22-month-old girl was born at 38 weeks of gestation, and she was the third child of healthy parents. Her birth weight was 2,714 g (10~25 centile), length was 46.5 cm (10~25 centile), and occipitofrontal circumference (OFC) was 34.1 cm (75~90 centile). Her father and mother were 37 and 35 years old, respectively. At birth, low-set ears were noted. From early infancy, she showed feeding difficulty because of hypotonia and hypersomnia. Early development was delayed, with head control at 5 months, rolling over at 8 months, sitting at 11 months, crawling at 13 months, and standing unsupported at 22 months. Brain magnetic resonance imaging, electroencephalography, and auditory brainstem response did not show any abnormalities.

At present, her height is 82.1 cm (10~25 centile), weight is 11.2 kg (50~75 centile), and OFC is 45.4 cm (10~25 centile). She has distinctive facial features, including midface hypoplasia, a flat nasal bridge, telecanthus, anteverted nares, a small nose, long philtrum, low-set and posteriorly rotated ears associated with hypoplastic ear cups, a right preauricular pit, thin lips, a high arched palate, and micrognathia. Arachnodactyly is also noted. Neurological examination reveals generalized hypotonia and right esotropia (Table 1).

**Table 1:** Clinical features of the patients w ith 11q12.3 duplication/triplication

		**Present patient**	**Tyson et al. (2005)**	**Jehee et al. (2007)**
			**(Subject 2)**	**(Patient 1)**
			**DECIPHER #253705**	
Physical				
	short stature	-	+	+
	microcephaly	-	+	+
	scoliosis	-	+	N A
	congenital heart defect	-	+	N A
	fingers	arachnodactyly	camptodactyly (5th finger)	brachydactyly
			brachydactyly (5th finger)	clinodactyly (5th finger
				broad thumbs
Face				
	upslanting palpebral fissure	-	+	-
	midface hypoplasia	+	NA	-
	flat nasal bridge	+	NA	-
	telecantus	+	NA	-
	anteverted nare	+	NA	-
	small nose	+	NA	-
	long philtrum	+	NA	-
	low-set and posteriorly rotated ears	+	NA	-
	preauricular pit	right	+	right
	high arched palate	+	NA	NA
	thin lips	+	NA	-
	micrognathia	+	NA	-
Neurological				
	developmental delay	+	+	+
	feeding difficulty	+	NA	NA
	hypotonia	+	+	NA
	hypersomnia	+	NA	NA
	static encephalopathy	-	+	NA
Examination				
	brain MRI abnormality	-	NA	NA
	EEG abnormality	-	NA	NA
	ABR abnormality	-	NA	NA

*MRI* magnetic resonance imaging; *EEG* electroencephalogram; *ABR* auditory brain response; *NA* not available.

## Results

**Figure 2:**
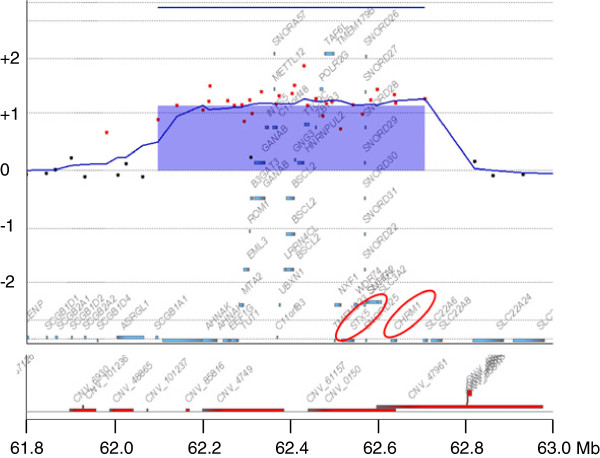
**The result of chromosomal microarray testing.** (Above) Gene View by Agilent Genomic Workbench (Agilent Technologies) is shown in horizontal view; the X- and Y-axes indicate genomic location and a log_2_ ratio of the intensity, respectively. The region of genomic copy number gain is shown by a blue-translucent rectangle with a mean log_2_ ratio of 1.132548 with the size of 557 kb in the 11q12.3 region. The gene symbols discussed in the text are emphasized by red circles. (Bottom) The locations of the known CNVs are depicted.

**Figure 3:**
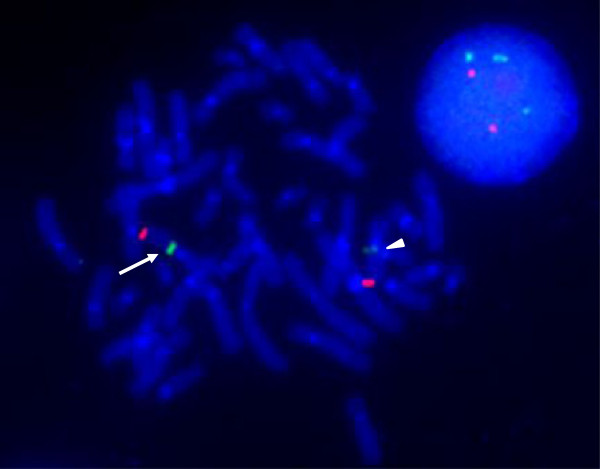
**FISH analysis by using BAC as the probes.** Red signals are markers of 11q25 labeled on RP11-469N6 (chr11:134,478,780-134,651,287) and green signals are targeted markers of 11q12.3 labeled on RP11-163K24 (chr11:62,514,291-62,691,426). In the interphase nucleus, 4 green signals are noted, confirming the triplication of the targeted region. One of the targeted green signals of the metaphase (arrow) is stronger than the other (arrowhead), indicating the triplication at the same chromosome 11.

The conventional G-banding technique showed a normal female karyotype of 46,XX. By use a 60K array, a gain of the genomic copy number was identified on the 11q12.3 region with a mean log_2_ ratio of 1.132548, indicating a triplicated segment of 557 kb with a description of arr 11q12.3(62,190,466-62,747,951)x4 (build 19) according to the International System for Human Cytogenetic Nomenclature (2013) (Figure 2). FISH analysis confirmed triplicated signals in the interphase nucleus (Figure 3). One of the targeted signals was stronger than the other on the metaphase spreads of FISH, indicating triplication in 1 of the 2 chromosome 11 homologues. Both parents’ samples showed no abnormalities by chromosomal microarray testing using 60K (data not shown), indicating de novo occurrence of this triplication.

## Discussion

**Figure 4:**
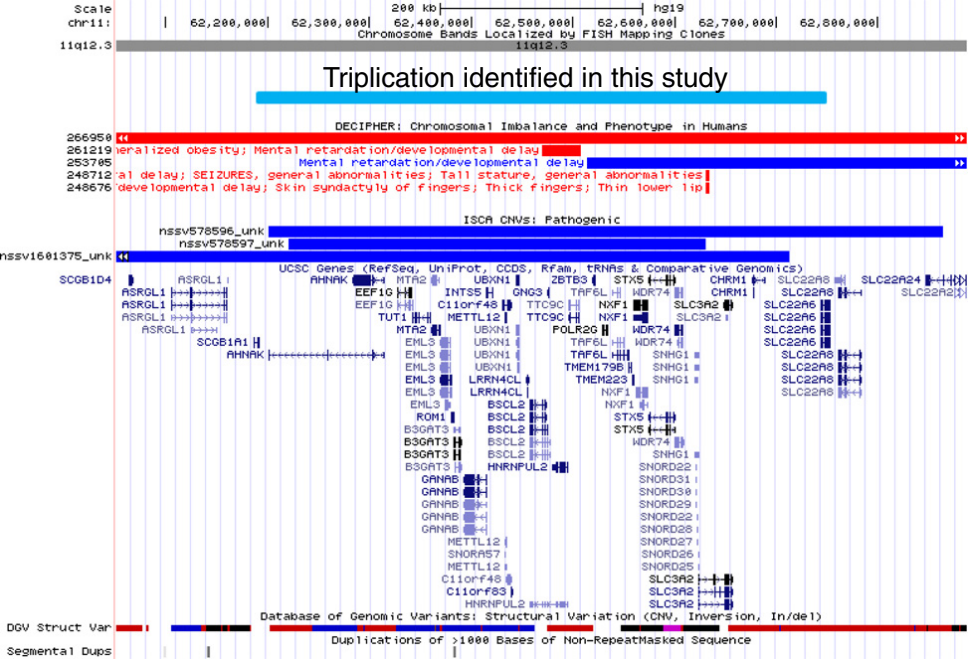
**Genomic map viewed using the UCSC genome browser.** Triplicated region of the patient (a light blue bar) is depicted on the UCSC genome browser. DECIPHER #253705 is indicated by a blue bar that shows the duplication previously reported by Tyson et al. Three overlapping duplications (blue bars) are included in the ISCA database.

Compared to the location of the triplication that resulted from DUP-TRP/INV-DUP, the triplicated region identified in the present study was not embedded within the duplicated segments and there was no duplicated region around the triplicated segment (Figure 2). Therefore, the breakpoints caused by this triplication would be the same among all fragments. Such small triplications that are not embedded within duplicated segments are rare, and we were able to identify similar cases in patients with Parkinson’s disease associated with triplications in the region of the synuclein alpha gene (*SNCA*) in the literature [9]. In such cases, triplicated regions, including *SNCA*, are not embedded within duplication. Because the triplicated region identified at 11q12.3 was quite small, we failed to confirm the insertion orientations of the triplicated segments using FISH analysis (data not shown). Due to the existence of many CNVs in this region (Figure 4), it was challenging to confirm the breakpoints of the triplications using polymerase chain reaction-based methods as mentioned in the introduction. Therefore, presently, the insertion orientations of the triplicated segments remain unknown.

Jehee et al. reported a patient with multiple craniosynostoses, a congenital heart defect, and developmental delay [10]. Cytogenetic analyses identified a mosaic existence of a large duplication of 11q11-q13.3 in that case. Because the duplicated region included the fibroblast growth factor 3 gene (*FGF3*) and fibroblast growth factor 4 gene (*FGF4*), Jehee et al. discussed the possibility that the gene dosage effects of these genes resulted in the onset of craniosynostosis [10]. Although the triplicated region identified in the present study was included in the duplicated region reported by Jehee et al. [10], the present patient did not show any symptoms of craniosynostosis and the candidate genes, *FGF3* and *FGF4*, were not included in the triplicated region because of the extremely small size of the aberrant region.

When we analyzed the International Standard for Cytogenomic Arrays (ISCA) database (https://www.iscaconsortium.org/), we found that 3 duplications were included in this region (Figure 4). Because the ISCA database includes no clinical information, we were unable to compare the phenotypic features of our patient with those of others; however, the existence of similar duplications in this region would suggest that the region may be prone to rearrangement as a result of its structural nature. The DECIPHER database (http://decipher.sanger.ac.uk/) showed only 1 overlapping duplication (chr11:62,514,284-65,140,178) in the 11q12.3 region in a patient (DECIPHER #253705). This patient had mild-to-moderate intellectual disability and other complications that had already been reported by Tyson et al. [11]. The common phenotypic features between our patient and the previously reported patients include brain developmental delay, finger abnormalities (including arachnodactuly, camptodactyly, brachydactyly, clinodactyly, and broad thumbs), and preauricular pits [10,11] (Table 1). Therefore, we believe that the genes responsible for brain developmental delay and the connective tissue abnormalities are located within the shortest region of overlap (SRO) (Table 2). With regard to the genes responsible for neurological features, including developmental delay and hypotonia, Tyson et al. listed the reticulon 3 gene (*RTN3*) as the candidate gene because of its highest level of expression in the brain [11]. However, *RTN3* is not included within the triplicated region in the present patient.

**Table 2:** The genes he genes included in the SRO

	**Gene Symbol**	**Function**
1	*ZBTB3*	zinc finger and BTB dom ain containing 3
2	*POLR2G*	polymerase (RNA) II (DNA directed) polypeptide G
3	*TAF6L*	TAF6-like RNA polym erase II, p300/C BP-associated factor (PCAF)-associated factor, 65kD a
4	*TMEM179B*	transmembrane protein 179B
5	*TMEM223*	transmembrane protein 223
6	*NXF1*	nuclear RNA export factor 1
7	*STX5*	syntaxin 5
8	*WDR74*	WD repeat dom ain 74
9	*SNHG1*	small nucleolar RNA host gene 1 (non-protein coding)
10	*SLC3A2*	solute carrier family 3 (activators of dibasic and neutral amino acid transport), member 2
11	*CHRM1*	cholinergic receptor muscarinic 1
12	*SLC22A6*	solute carrier family 22 (organic anion transporter), member 6

Alternatively, the syntaxin 5 gene (*STX5*) and the cholinergic receptor muscarinic 1 gene (*CHRM1*) (Table 2), in the SRO may be related to the neurological features of these 2 patients because of their functional relevance to the central nervous system. *STX5* encodes a member of the syntaxin or t-SNARE (target-SNAP receptor) family [12]. These proteins are found on cell membranes and serve as targets for v-SNAREs (vesicle-SNAP receptors), permitting specific synaptic vesicle docking and fusion [13,14]. *CHRM1* is reported to have some relevance to neuronal functions. Muscarinic receptors regulate several effects of acetylcholine in the central and peripheral nervous system. The results of *CHRM1*-null mutant mice investigations suggested that the M1 receptor is specifically involved in memory processes for which the cortex and hippocampus interact [15]. Additionally, the functional relevance of *CHRM1* in schizophrenia and depressive disorders has been suggested [16,17].

Since the 1998 review that coined and defined the term “genomic disorders” [18], a multitude of genomic disorders caused by genomic rearrangements have been identified [19], and many of them manifest neurological features, including mental impairments, autistic features, and psychiatric disorders [20,21]. This finding is because there are many dosage-sensitive genes related to nervous system functions. Additionally, there is disputable evidence that CNVs can play a role in the pathogenesis of neurodevelopmental and neurodegenerative disorders [22]. In the present study, we proposed potential candidate genes for developmental delay; however, it is difficult to determine which genes contribute to the connective tissue involvement, including distinctive facial findings, finger abnormalities, and pre-auricular tags seen in the patients with genomic copy number gains of 11p12.3.

## Methods

Blood samples were collected upon approval of the ethics committee of our institution. A patient’s karyotype was analyzed using the conventional G-banding technique at 400–500 band resolution. Genomic DNA was extracted from blood samples using QIAamp DNA extraction kit (QIAGEN, Hilden, Germany) and was subsequently evaluated. Chromosomal microarray testing was performed using Agilent Human Genome microarray 60K (Agilent Technologies, Santa Clara, CA), as described previously [23]. Extracted data was analyzed using Agilent Genomic Workbench ver. 6.5 (Agilent Technologies). Metaphase spreads prepared using the patients’ blood samples were used for FISH analyses to confirm the results of chromosomal microarray testing. The bacterial artificial clones (BAC), RP11-469N6 and RP11-163K24, were selected from the UCSC genome browser (http://genome.ucsc.edu/) for use as probes. Both parental samples were obtained and analyzed using chromosomal microarray testing with a 60K array to confirm whether the aberration identified on the patient was de novo.

## Consent

Written informed consent was obtained from the patient’s family.

## Competing interests

None of the authors has any conflict of interest to disclose.

## Authors’ contributions

TY constructed this research, analyzed and interpreted chromosome microarray data, considered the genotype-phenotype correlation, and drafted the paper. SS, NS, and KSh performed the cytogenetic studies. MM and SA correlated clinical findings. KSa supervised and reviewed this article. All authors read and approved the final manuscript.
